# Influence of surface chemistry and morphology of nanoparticles on protein corona formation

**DOI:** 10.1002/wnan.1788

**Published:** 2022-03-07

**Authors:** Roberta Bilardo, Federico Traldi, Alena Vdovchenko, Marina Resmini

**Affiliations:** ^1^ Department of Chemistry Queen Mary University of London London UK

**Keywords:** biocorona, nanoparticles, protein corona

## Abstract

Nanomaterials offer promising solutions as drug delivery systems and imaging agents in response to the demand for better therapeutics and diagnostics. However, the limited understanding of the interaction between nanoparticles and biological entities is currently hampering the development of new systems and their applications in clinical settings. Proteins and lipids in biological fluids are known to complex with nanoparticles to form a “biomolecular corona”. This has been shown to affect particles' morphology and behavior in biological systems and their interactions with cells. Hence, understanding how nanomaterials' physicochemical properties affect the formation and composition of this biocorona is a crucial step. This work evaluates existing literature on how morphology (size and shape), and surface chemistry (charge and hydrophobicity) of nanoparticles influence the formation of protein corona. The latest evidence suggest that although surface charge promotes the interaction with proteins and lipids, surface chemistry plays a leading role in determining the affinity of the nanoparticle for biomolecules and, ultimately, the composition of the corona. More recently the study of additional nanoparticles' properties like shape and surface chirality have demonstrated a significant effect on protein corona architecture, providing new tools to tailor biomolecular corona formation.

This article is categorized under:

Therapeutic Approaches and Drug Discovery > Emerging Technologies
Toxicology and Regulatory Issues in Nanomedicine > Toxicology of Nanomaterials

## Graphical abstract

The surface chemistry and morphology of nanoparticles influence their interfacial behavior, in particular with biological entities like proteins and lipids. Both quantity and composition of the biomolecular corona can be tailored by carefully designing nanomaterials with specific characteristics, determined by the targeted application.

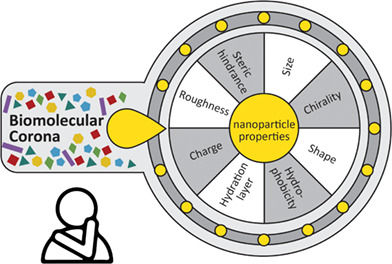

## 1 INTRODUCTION

Nanomaterials play an increasingly important role in medicine, providing tools for diagnosing, treating, and preventing diseases, with drug delivery being one of the areas that have attracted considerable research. The development of nanoparticles (NPs) for the delivery of pharmaceutically active compounds to the body raises important questions regarding their behavior in vivo, and more specifically their interactions with bio‐interfaces and biological fluids. One of the challenges that need to be addressed, when developing NPs for drug delivery, is their interaction with proteins and other biological molecules present in the blood (Barui et al., 2020; Digiacomo et al., 2020; Min et al., 2021; Sharifi et al., 2020; Xiao & Gao, 2018). The term “protein corona” (PC) is used to describe the layer that proteins form around NPs when placed in a biofluid (Liu, Tang, & Ding, 2020); the term is further classified into “hard corona,” identifying proteins irreversibly attached to NPs, and “soft corona,” for proteins loosely attached to the nanomaterial (Docter et al., 2015; Liu, Tang, & Ding, 2020). The analytical methods for the evaluation of “soft” and “hard” PC has been extensively reviewed (Pederzoli et al., 2017). More recently the term “bio‐corona” has been introduced, to reflect the wider pool of bio‐systems that NPs have access to, and that can result in significant changes to their biological activity, with positive or negative outcomes (Stater et al., 2021).

**FIGURE 1:**
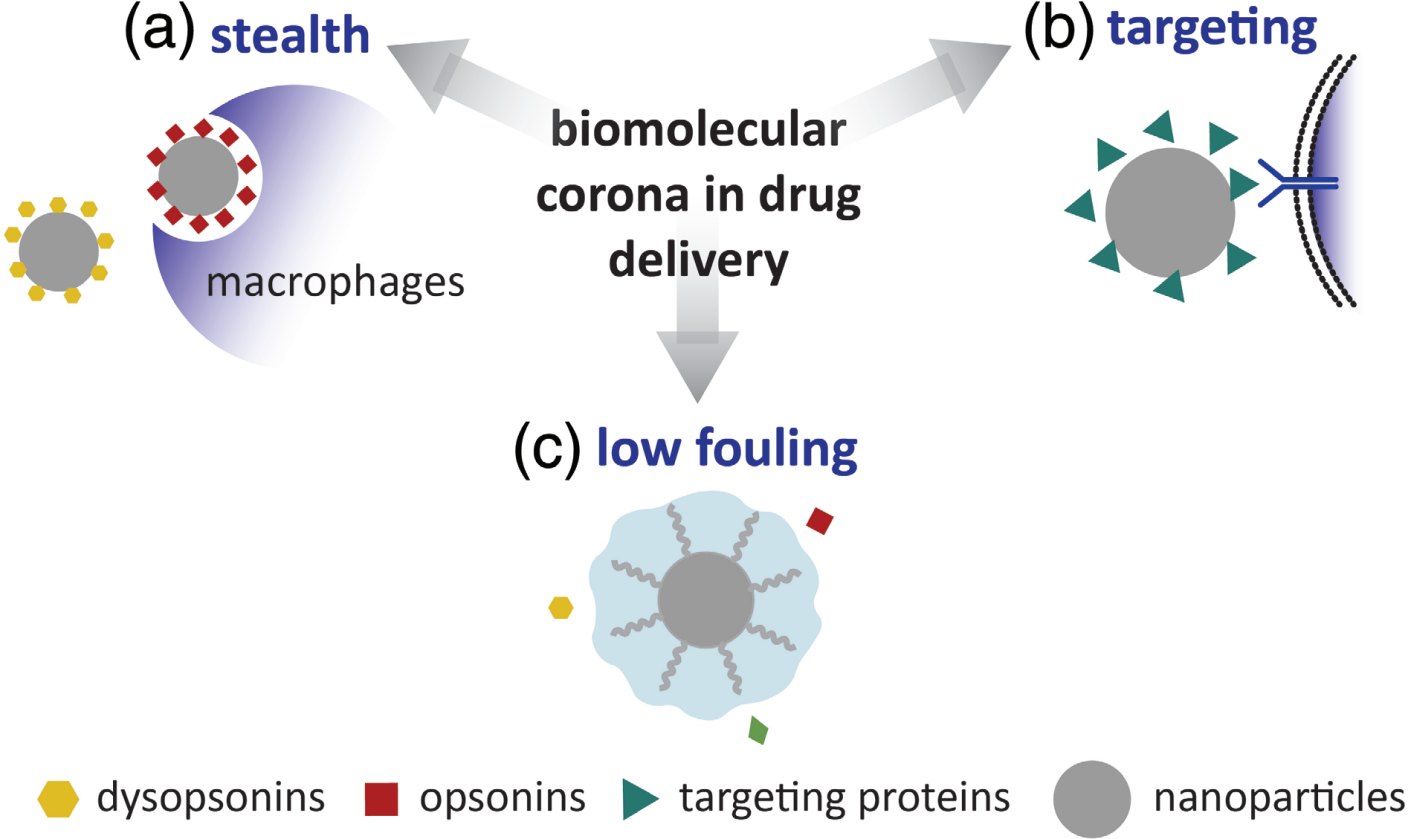
Schematic representation of the different approaches to protein corona (PC) when developing drug delivery systems

Many different nanomaterials have been reported to strongly interact with biological fluids and form a bio‐corona, (Galmarini et al., 2018; Mandal et al., 2018; Tenzer et al., 2013; Yu et al., 2020), and this property has led to important applications, for example, as an analytical tool for the detection of different diseases (Giulimondi et al., 2019), such as cancer (Di Domenico et al., 2018). However, the formation of bio‐corona is not always a desirable property: for example, it was shown that NPs without PC has higher targeting efficiency (Feiner‐Gracia et al., 2017; Md Rasib et al., 2019) and cellular uptake (Breznica et al., 2020; Ghazaryan et al., 2019; Giulimondi et al., 2019.; Simon & Morsbach, 2018; Tonigold et al., 2018). The study of biomolecular corona, in the context of drug delivery, has been used by researchers mainly to tailor the properties of NPs for three main objectives, schematically shown in Figure 1: (i) to introduce “stealth” characteristics, (ii) to have efficient targeting, and (iii) to obtain low fouling materials.

“Stealth” properties refer to the ability of NPs to avoid detection by the immune system, resulting in enhanced circulation in vivo, through the adsorption of dysopsonins (Figure 1a). It has been shown that the coating of NPs with polyethylene glycol (PEG) or zwitterionic ligands (Srivastava et al., 2020; Yang et al., 2020) results in the enrichment of these proteins, as opposed to opsonins, on the particle's surface, reducing cellular uptake by macrophages (Papini et al., 2020). The physicochemical properties of the NPs' surface can determine the fate of the nanomaterial in vivo; for example, apolipoprotein H (ApoH), a type of dysopsonin, was found enriched on the negative surface while opsonin immunoglobulin G (IgG) preferentially adsorbed on surfaces bearing stronger basic functionality (Luo et al., 2017). A nanomaterial designed to favor the preferential adsorption of dysopsonins in vivo, is one of the approaches to control PC. Alternatively, stealth properties can be introduced to a NP by using a preformed PC (Giulimondi et al., 2019; Mackiewicz et al., 2019; Simon & Morsbach, 2018), nanomaterials are incubated in a dysopsonin‐enriched medium and are then directly injected into the bloodstream.

The second approach focuses on using the PC for targeting purposes (Figure 1b). Several recent publications have reported that the adsorption of apolipoproteins on the NP surface increases their transport across the blood–brain barrier (Min et al., 2021; Simon & Morsbach, 2018; Zhang et al., 2019). Similarly, it was shown that the preliminary adsorption of specific antibodies on the surface of nanomaterials results in enhanced targeting (Tonigold et al., 2018).

The third approach focuses on developing nanomaterials with low‐fouling properties, designed to reduce PC formation as much as possible (Figure 1c). There is extensive literature covering this particular aspect, showing, for example, that highly hydrophilic and zwitterionic coatings lead to low‐fouling properties (Alberg et al., 2020; Almalik et al., 2017; Johnston et al., 2017; Li et al., 2019; Zhang et al., 2013) and that physicochemical properties of nanomaterials can be used to achieve low‐fouling in a variety of nanomaterials.

Overall, control of PC formation is highly dependent on the study and understanding of the relationship between physicochemical characteristics of NPs and their interaction with proteins. The correlation between properties of nanomaterials and adsorption of proteins has been reviewed extensively (Kharazian et al., 2016; Li et al., 2020; Mahmoudi et al., 2013; Walkey & Chan, 2012; Xiao & Gao, 2018), however, the advances in knowledge and significant technological progress are contributing to the understanding of the role played by the NPs' structural features. In this review, we highlight recent studies on the physicochemical properties that allow to control and tailor PC formation, in particular focusing on surface chemistry, morphology, and surface roughness.

## 2 PROTEIN CORONA AS A FUNCTION OF NANOPARTICLE SURFACE CHEMISTRY

PC formation relies significantly on the interfacial behavior of NPs with the protein's outer layer, and therefore the surface chemistry and physical properties of the materials play an important role. The next section will provide an overview of the latest advances in this area, focusing on hydrophobicity, steric hindrance and chirality, and surface charge.

### 2.1 Hydrophobicity

When studying PC formation, hydrophobicity is among the most widely investigated chemical properties. Both organic and inorganic particles, with varying degrees of hydrophobicity, have been studied, to evaluate the effect of these variations on PC formation. Table 1 presents an overview, using a selection of literature data.

**TABLE 1:** Overview of studies focusing on the impact of hydrophobicity on the corona formation around different types of nanomaterials

	Core material	Surface chemistry	Protein/media	References
Inorganic Core	Gold	Alkane Ethylene glycol	FBS	(Yu et al., 2020)
		Citrate Glycan PEG	HSA	(Meesaragandla et al., 2020)
		Alkyl ligands	HS	(Saha et al., 2016)
		Methyl Hydroxyl	ApoE, IgE, HSA	(Lu et al., 2019)
		Cetrimonium bromide Poly(styrene sulfonate) Poly(diallyldimethylammonium chloride) Poly(ethylene imine) PEG	HP	(Cai et al., 2020)
	Silica	Pristine	HP	(Monopoli et al., 2011)
		Pristine Amine Carboxyl	HP	(Tenzer et al., 2013)
	Graphene sheets	Pristine Hydroxyl	ApoE, IgE, HSA	(Lu et al., 2019)
Mixed core	Iron–platinum/poly(maleic anhydride‐alt‐dodecene)	PEG	HSA, Fib	(Pelaz et al., 2015)
	CdSe–ZnS quantum dots/dihydrolipoic acid	PEG Zwitterionic	BSA, FBS	(Perng et al., 2019)
	Magnetite/PLGA Magnetite/Dextran Magnetite/phosphatidylcholine	Carboxyl PEG Phosphatidylcholine	HP, porcine pulmonary surfactant	(Raesch et al., 2015)
Organic Core	PLGA	Pristine PEG	FBS	(Partikel et al., 2019)
	HSA PLGA/Didodecyldimethyl ammonium bromide	Pristine PEG	FBS	(Gossmann et al., 2015)
	Polystyrene	Pristine Amine Carboxyl	HP	(Tenzer et al., 2013)
		Pristine PEG PPE	HP	(Simon et al., 2018);
		Pristine PPE	HSA	(Müller et al., 2017)
		Sulfonate	HP	(Monopoli et al., 2011)
	Polystyrene Methylstyrene, *Tert*‐butylstyrene Methylmethacrylate Acrolein	–	HP	(Gessner et al., 2000)
	NIPAM/BAM	–	HP, HSA, Fib	(Cedervall et al., 2007)

Abbreviations: ApoE, apolipoprotein E; BSA, bovine serum albumin; CdSe‐ZnS, cadmium selenide zinc sulfide; FBS, fetal bovine serum; fib, fibrinogen; HP, human plasma; HS, human serum; HSA, human serum albumin; IgE, immunoglobulin E; NIPAM/BAM, N‐isopropylacrylamide/N‐*tert‐*butylacrylamide; PEG, poly(ethylene glycol); PLGA, poly(d,l‐lactide‐co‐glycolide); PPE, poly(phosphoester).

#### 2.1.1 Hydrophobic character of nanoparticles drives protein corona formation

In a pioneering study, the impact of progressively increasing the degree of hydrophobicity in organic N‐isopropylacrylamide:N‐*tert‐*butylacrylamide (NIPAM:BAM) copolymers on PC formation was reported (Cedervall et al., 2007). The authors found that by increasing the ratio of hydrophobic BAM monomer in the polymer, from 15% to 50%, the quantity of proteins bound to the NPs' surface increased by 5‐fold. In a similar work, the degree of hydrophobicity of inorganic gold NPs was gradually increased by surface functionalization with different ligands. The more hydrophobic NPs attracted double the amount of proteins on their surface, compared to the more hydrophilic ones (Yu et al., 2020). The greater tendency of hydrophobic NPs to attract more proteins has been reported mainly with particles of similar chemical composition. However, when different types of NPs (such as inorganic and organic) were investigated, hydrophobicity was not always the main driving force for PC formation. For instance, Monopoli et al. (2011) found lower PC formation on hydrophobic polystyrene NPs, compared to hydrophilic silica ones. This apparently inconsistent result is closely correlated to the different mechanisms of interactions of the two types of NPs with proteins; while proteins were adsorbed via hydrophobic forces in the case of polystyrene NPs, H‐bonding, and electrostatic interactions were prevalent in the case of silica NPs. This study suggests that although hydrophobicity generally enhances NP‐protein interactions, it cannot be considered as an absolute parameter in determining the PC, which is the result of the interplay of different factors instead.

Despite the highlighted importance of hydrophobic forces in the formation of PC, the absorption of more proteins on hydrophobic nanomaterials compared to hydrophilic ones is not necessarily evidence of the prevalence of hydrophobic proteins. Proteins, in general, are characterized by the co‐presence of hydrophobic and hydrophilic domains which, in the presence of nanomaterials, can rearrange spatially to maximize the interaction with the nanomaterial's surface, assuming a more hydrophobic/hydrophilic character (Mukhopadhyay et al., 2018). These conformational changes, which can affect the secondary and/or tertiary structure of proteins, are thermodynamically driven and can eventually lead to the protein unfolding and consequent denaturation (Chatterjee et al., 2010). Using computational modeling, Lu and co‐workers investigated the adsorption behavior of different proteins on graphene sheets and gold NPs (Lu et al., 2019). The lack of a direct correlation between the overall character of the investigated proteins and their affinity toward specific types of NPs was confirmed by the observed behavior of human serum albumin (HSA). Although this protein generally assumes hydrophilic conformations (Retnaningtyas et al., 2016), its absorption was lowered by the presence of a higher number of hydroxyl groups on the nanomaterials. Similarly, Cedervall et al. found an enhanced adsorption of structurally different proteins including HSA, fibrinogen, and apolipoprotein A‐I (ApoA‐I) on more hydrophobic NIPAM:BAM copolymers, suggesting the secondary importance of the initial native protein conformation (Cedervall et al., 2007). Nevertheless, some proteins have been found to prefer binding more hydrophobic or hydrophilic NPs highlighting that the presence of a specific sequence of amino acids can favor some interactions overall. These preferential bindings, resulting from the intrinsic more flexible nature of proteins, have important effects on the final fate of the NPs in vivo, as discussed more in detail in Section 2.1.3.

#### 2.1.2 Surface modifications allow to tailor protein corona formation

PC can change significantly the behavior of NPs in vivo, therefore its control has been extensively studied to facilitate applications of NPs in nanomedicine. One of the main strategies to reduce the formation of the corona has featured the functionalization of particles with hydrophilic ligands and coatings (Amoozgar & Yeo, 2012; Ke et al., 2017; Rampado et al., 2020). Functionalization with hydrophilic motifs promotes the formation of hydration shells, thus making the NP surface less available for interactions with proteins and other biomolecules.

Among the potential ligands, PEG is certainly the most well‐known and used surface motif. Although PEG chains reduce NP‐protein interactions thanks to their hydrophilic character, other properties such as polymer chain length and density on the particle surface have also been found to play an important role. The effect of coating poly(lactic acid) (PLA) NPs with PEG ligands ranging from 2 to 20 kDa on PC formation was reported over two decades ago (Gref et al., 2000). A strong decrease in protein adsorption was initially observed when particles were functionalized with PEG ranging between 2 and 5 kDa. Only a small reduction was then seen for PEG chains longer than 5 kDa, suggesting that the optimum NP coverage had been reached at 5 kDa. In a different study, polymeric micelles coated with PEG ligands and incubated in whole human blood showed a progressive inhibition of PC formation when PEG size increased from 5 to 20 kDa, indicating that the increase in molecular weight of the PEG chains results in a decrease in the formation of PC (Miteva et al., 2015). Interestingly, the two studies also indicate that for different particles systems, the optimal length of the PEG chains might have to be tailored. Nevertheless, coating of NPs with PEG does not always reduce the PC formation efficiently. As described by Seneca et al., the amount of proteins adsorbed on PEGylated NPs varied significantly depending on the density of the PEG chains present on the surface (Seneca et al., 2018). This study, together with other similar data present in literature (Perry et al., 2012; Walkey et al., 2012; Zhang et al., 2015), suggests that highly dense PEG layers prevent the corona formation more efficiently. The adsorption of proteins on low dense PEGylated particles was also investigated by Pelaz et al. In their study, the authors focused on the corona formed around PEGylated polymer‐coated iron NPs finding that model proteins HSA and fibrinogen were able to penetrate in between the loosely packed PEG chains and to adsorb on the NP surface (Pelaz et al., 2015).

**FIGURE 2:**
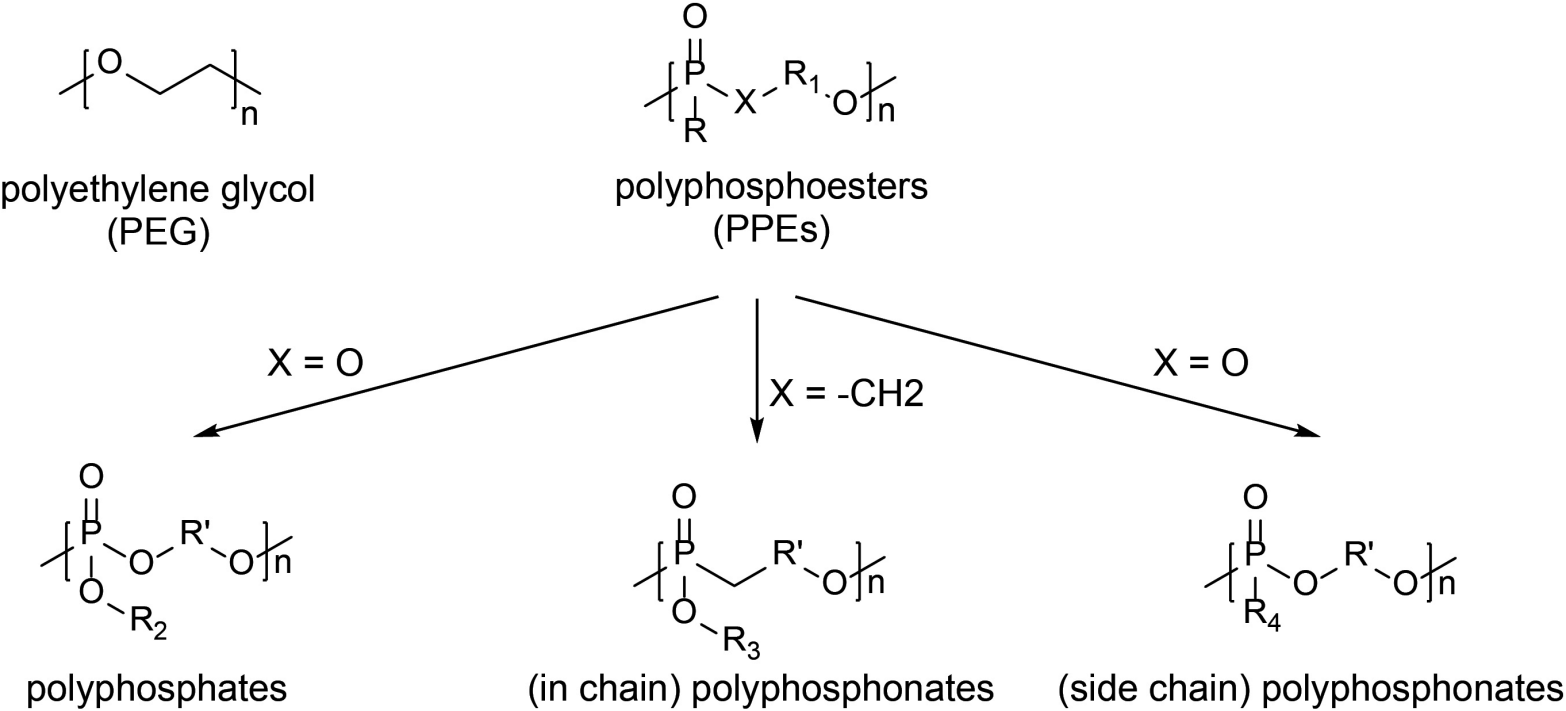
Chemical structures of PEG and PPE‐based ligands with different hydrophilicity

Despite its efficient reduction in the adsorption of proteins, the use of PEG has been recently questioned because of some limitations. These include (1) its nonbiodegradable character in vivo, leading to both its accumulation and uncontrolled oxidative degradation into toxic products (Yamaoka et al., 1994); (2) the triggering of specific anti‐PEG antibodies with the consequent danger of hypersensitivity reactions (Yang & Lai, 2015); (3) unspecific reduction of cellular uptake, causing issues to access target cells (Zhang et al., 2015). Therefore, other classes of stealth ligands with less limitations have been investigated; among these polyphosphoesters (PPEs) seem to be very promising candidates (Pelosi et al., 2020). Compared to bare particles, polystyrene NPs functionalized with different PPE derivatives showed the decreased formation of PC to a level comparable to PEG (Müller et al., 2017; Simon et al., 2018). More recently, in a study by Bauer et al., functionalization of poly(methyl methacrylate) and bio‐based hydroxyethyl starch nanocarriers with PPE ligands having different degrees of hydrophilicity (Figure 2) revealed the versatility of these ligands (Bauer et al., 2020). Interestingly, different types of PPE coatings have led to the enrichment of certain proteins in the corona, encouraging the use of these ligands as tools to promote changes in the PC composition.

As a more biocompatible alternative to PEG, glycan‐based coatings have also been used to reduce PC formation. These polysaccharide coatings have been found to decrease the amount of proteins adsorbed on different types of materials, such as gold NPs (Henriksen‐lacey & Grzelczak, 2015) and carbon surfaces (Zen et al., 2016). Nevertheless, when compared to PEGylated particles, Meeseragandla et al. found that glycan‐coated NPs adsorbed a greater amount of HSA molecules (Meesaragandla et al., 2020). These NP‐protein interactions were mostly attributed to the formation of hydrogen bonds, promoted by the presence of hydroxyl groups within the glycan structure, therefore questioning the efficiency of these ligands in preventing corona formation. This result is consistent with the criteria for anti‐fouling materials, proposed by Ostuni et al., which should not have hydrogen bond donor groups, such as hydroxyl units found in glycans (Ostuni et al., 2001). Although the use of hydrophilic polymer coatings such as PEG and PPE has been endorsed by the scientific community as a standard approach to limit the formation of a PC, more precise information regarding the mechanisms of interaction of proteins with these types of coatings is still required. This knowledge may enable the design of more efficient coatings able to prevent protein adsorption on nanomaterials and may improve the biosafety of future nano‐based therapeutics.

#### 2.1.3 Protein corona formation: A positive tool for in vivo applications

The hydrophilic/hydrophobic character of NPs affects the formation of the PC not only in terms of the quantity of proteins but also in overall composition. This influence is particularly important for the behavior of NPs in vivo, as it is strictly correlated to their biological fate (Zhu et al., 2013). In fact, NP's properties such as bioavailability and uptake by phagocytic cells have been shown to depend on the preferential adsorption of specific proteins. These proteins can either favor the endocytic incorporation (opsonins) or inhibit this action and prolong the in vivo circulation time of NPs (dysopsonins; Owens & Peppas, 2006). NP's hydrophobicity has been linked to the adsorption of specific opsonins. A study featuring molecular dynamic simulations (MD) performed on graphene and gold nanomaterials showed that the binding of immunoglobulin E (IgE) increased with the nanomaterials' hydrophobicity (Lu et al., 2019). A similar trend was also observed for other opsonins such as fibrinogen (Schöttler et al., 2016), hemoglobin fetal subunit beta (Yu et al., 2020), and complement factors (Partikel et al., 2019). All these studies agree that the interaction between these proteins and NPs is mainly driven by hydrophobic forces. On the other hand, the adsorption of dysopsonins is favored by the presence of hydrophilic ligands on the NP surface. In 2016, Schöttler et al. observed a significant enrichment of apolipoprotein J (ApoJ, or clusterin) on the corona of polystyrene NPs functionalized with PEG and PPE ligands (Schöttler et al., 2016), and a consequent reduction of the cellular uptake of more than 75% which confirmed the stealth effect due to the presence of this protein. Similarly, another efficient dysopsonin, apolipoprotein C3, was exclusively detected on the corona of poly(d,l‐lactide‐co‐glycolide (PLGA) NPs after their functionalization with PEG (Partikel et al., 2019). These results highlight that the efficiency of PEG and other stealth coatings in reducing the NPs clearance by the immune system is the result of both reductions of the protein adsorption and enrichment in dysopsonins. However, the preferred affinity of apolipoproteins, which mostly act as dysopsonins, for more hydrophobic or hydrophilic NPs is still the subject of debate. In fact, given their function in binding lipids and transporting them through the bloodstream, they are characterized by both hydrophobic and hydrophilic domains and are able to interact with a wide range of NP surface chemistries (Ritz et al., 2015). Overall, these results provide important evidence that the composition of the PC and, thus, its effect on the behavior of NPs in vivo may be affected by the chemical composition of the nanomaterial surface. This information might be used to develop nanomaterials with reduced (or enhanced) recognition by the immune system and might offer the opportunity to design more efficient nanomedicines.

### 2.2 Steric hindrance and chirality

**FIGURE 3:**
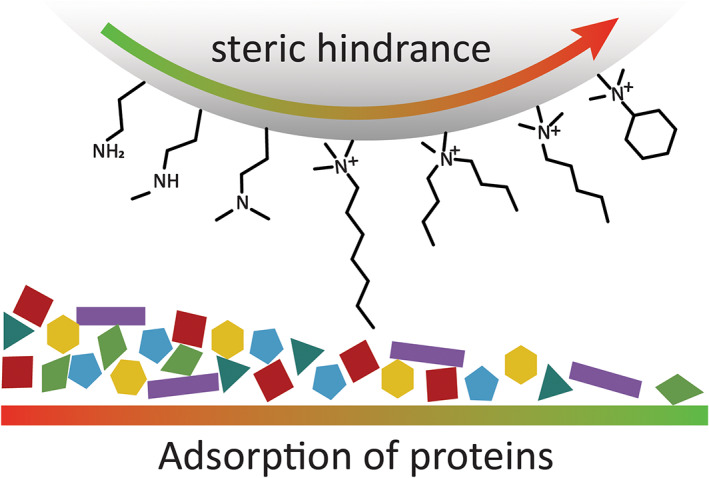
Schematic summary of the impact of differently functionalized amino groups on the NP surface and their impact on the protein corona formation

The presence of hydrophobic and hydrophilic groups is not the only factor influencing the adsorption of proteins on NPs, in fact, multiple studies have highlighted the importance that steric hindrance and chirality of surface units can have not only in promoting protein adsorption but also favoring the interaction with specific categories of proteins (Figure 3).

Saha et al. reported on the impact of steric hindrance by using a series of surface motifs characterized by similar hydrophobicity, but different spatial arrangements (Saha et al., 2016). The data showed an interesting correlation between the amount of proteins adsorbed on the NPs surface and the bulkiness of the units; by comparing ligands with the same number of carbon atoms but with a different distribution, the team demonstrated that protein adsorption is overall less promoted by the presence of bulkier terminal groups such as branched or cyclic terminal units. Furthermore, differently distributed terminal groups corresponded to different compositions of the corona, resulting in the enrichment of different types of proteins.

In 2018, a similar approach was reported by Burnand et al., who evaluated the impact of different amino groups on the types of proteins adsorbed on gold NPs (Burnand et al., 2018). Four types of poly(vinyl alcohol‐co‐N‐vinylamine) copolymers, containing primary, secondary, or tertiary amines, were used to functionalize gold NPs, and the resulting PC composition was evaluated via liquid chromatography‐tandem mass spectrometry. Focusing on the relative presence of four proteins in the corona of the different NPs, this study showed that serum albumin and alpha‐2‐HS‐glycoprotein were more prevalent in the coronas of NPs functionalized with primary amines, whereas hemoglobin fetal subunit beta and hemoglobin subunit alpha interacted more with secondary and tertiary amines. This result suggests that ligands with different bulkiness may partially limit the interactions between the amine group on the particle surface and the polar amino acids on specific proteins (Gessner et al., 2000). Although at present only a limited amount of work has focussed on elucidating the role of amine bulkiness, these interesting results suggest that even subtle changes in ligands structures may influence the composition of the PC.

The hypothesis that subtle changes in ligand structure may affect the PC has attracted considerable interest, and recently led to studies on the role of surface chirality on PC. Although evidence of stereoselective interactions occurring between chiral nanointerface and biomacromolecules was reported by Zhang et al. already in 2012 (Zhang et al., 2012), studies of the effects of nanochirality on PC have been limited by several challenges. Among these, the most relevant is the lack of precise synthetic strategies for the preparation of homogenous chiral nanomaterials (Utembe, 2019) and the need for highly sensitive techniques able to evaluate the response of biological systems to nanochirality. Most of the studies performed so far have focussed on the evaluation of the effect of chirality on the interaction with a single type of protein. Qu et al. investigated indium phosphide zinc sulfide (InP@ZnS) quantum dots (QDs), functionalized with either d‐ or l‐penicillamine (d‐ or l‐Pen), in the presence of HSA (Qu et al., 2020). Fluorescence resonance energy transfer (FRET) was exploited to quantitatively analyze the protein binding behavior, determining the parameters of an adapted Hill equation (Del Pino et al., 2014). The lowest apparent dissociation constant (*K*_D_) observed in the case of l‐Pen‐QDs indicated their stronger capability to bind HSA, compared to d‐Pen‐QDs, confirming the impact of chirality of surface ligands on the binding of proteins. The same QDs chemically modified with either l‐ or d‐cysteine (l‐ or d‐Cys), were analyzed in order to evaluate the specificity of their interaction with HSA. Consistently with the penicillamine‐bearing particles, l‐Cys‐QDs showed stronger interactions with HSA compared to d‐Cys‐QDs. However, the observation of a less closely packed corona around d‐Cys‐QDs highlighted that different chiral centers may lead to different conformational rearrangements of proteins bound to the particles' surface. These results were a good proof of concept, suggesting that the impact of chirality on protein adsorption is closely dependent on the type and chemistry of NPs and the choice of biomolecules. Another interesting work in this area is the study of how the two chiral forms of penicillamine, used to functionalize gold NPs, interact differently with bovine serum albumin (BSA; Wang et al., 2018). Dynamic light scattering, quartz crystal microbalance with dissipation monitoring, fluorescence, zeta‐potential, circular dichroism, and infrared spectroscopy, were used to demonstrate that l‐Pen functionalized NPs displayed a stronger affinity for BSA, in agreement with the trend observed with QDs. Interestingly, the different spatial orientation of the Pen ligands caused BSA to bind in a different way to the NP surface; in particular, contact sites, adsorption orientation, and binding strength of interaction were all affected by the chirality of the particles. These results suggest that this property could be used not only to limit the adsorption of proteins, but also to control their orientation with important implications on cellular recognition.

Although most of the studies on the influence of chirality on PC focus on NPs interacting with single proteins, exposure to a mixture of proteins is the next step, to evaluate the impact on the PC composition. In a study performed by Piloni et al., polymers functionalized with different sugar epimers and incubated in presence of fetal bovine serum (FBS) showed different enrichment of some proteins in their corona (Piloni et al., 2019). These differences were considered evidence of the impact of the different sugars on nonspecific protein‐binding. In fact, given that the investigated NP types differed only for the spatial distribution of hydroxyl groups on their surface, their different affinities toward certain proteins revealed the dependence of NP–protein interactions from the specific localization of these groups on the surface.

The PC patterns observed in the presence of ligands with different steric hindrance or chirality demonstrate the relevance of surface chemical properties in NP‐protein interactions. Given that chiral surfaces differ for the spatial distribution of specific functional groups, identifying the contact sites is not a simple task and requires a close interconnection between experimental evidence and computer simulations. Even though the development of more specialized techniques has recently allowed to investigate the impact of chirality more effectively, the overall interaction mechanisms are not clear yet and each experimental result has to be strictly contextualized within the specific systems investigated and conditions used.

### 2.3 Surface charge

Proteins' adsorption to NPs is often dependent on properties such as size, hydrophobicity, chemical structure, and surface charge (Duan et al., 2020; Tenzer et al., 2013). The latter has been studied extensively and there is evidence of some correlation between particle charge, as determined by the zeta potential value, and the degree of PC formed. Zeta‐potential is a parameter that is used to identify the overall charge for NPs, however it does not give an information on the local distribution of charges. This section provides an overview of the effects of surface charges on the adsorption of proteins on NPs, highlighting recent findings on charged nanomaterials with low‐fouling characteristics and the relationship between surface charge and in vivo behavior, which is correlated with PC formation.

#### 2.3.1 Positively versus negatively charged NPs

**FIGURE 4:**
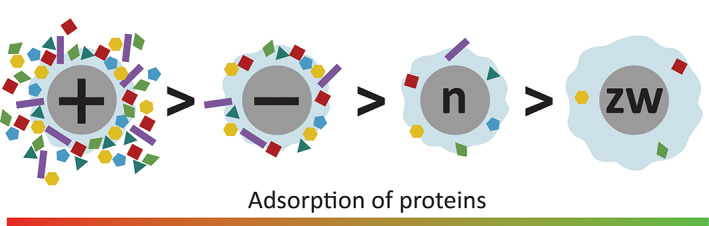
Schematic representation of findings, regarding the comparison between the amounts of adsorbed proteins depending on the charge of pristine nanomaterial, summarized in Table 2

The impact of charges in driving PC formation on NPs has been widely reported for a range of materials (Abstiens et al., 2019; Giulimondi et al., 2019; Gräfe et al., 2019; Meesaragandla et al., 2020; Mosquera et al., 2020; Sakulkhu et al., 2015; Srivastava et al., 2020; Yang et al., 2020) Table 2 provides an overview of the results, detailing the type of NP, the size and the media used, as a source of proteins. The overall trend is schematically depicted in Figure 4.

**TABLE 2:** A summary of findings, regarding the comparison between the amounts of adsorbed proteins depending on the charge of pristine nanomaterial

NPs type	NPs size	Protein/media	Adsorption of proteins	References
Inorganic NPs with polymer coating	10, 50 nm	Fetal calf serum	+ > − > zw	(Gräfe et al., 2019)
	13–20 nm	Human serum albumin	+ > −	(Meesaragandla et al., 2020)
	<150 nm	Fetal bovine serum	+ > −	(Sakulkhu et al., 2015)
	<40 nm	Human plasma	+ > n > zw	(Srivastava et al., 2020)
	15 nm	Fetal bovine serum	− > zw	(Mosquera et al., 2020)
Core–shell polymer NPs	60–70 nm	Fetal calf serum	+ > other	(Abstiens et al., 2019)
Liposomes	100–200 nm	Fetal bovine or human serum	charged > n	(Yang et al., 2020)
	100–200 nm	Human plasma	+ > −, n	(Giulimondi et al., 2019)

There is agreement in literature data that positively charged NPs consistently attract higher quantities of proteins, when placed in biological media, independently from the type of material used (inorganic, polymeric core–shell NPs, liposomes); this is a result of strong electrostatic interactions between the NPs and the blood proteins, that mainly have an overall negative zeta‐potential (Docter et al., 2015). The experimental data, however, clearly suggest that NPs with a negative zeta potential also attract high amount of proteins, due to electrostatic interactions with cationic patches in blood proteins (Giulimondi et al., 2019; Gräfe et al., 2019; Meesaragandla et al., 2020; Sakulkhu et al., 2015). Biological media is characterized by high ionic strength, therefore, long‐distance electrostatic forces can be shielded in such environment (Treuel et al., 2014). This explains why the adsorption of proteins in vivo does not always correlate with zeta potential of NPs and proteins, since short‐distance forces between charged functionalities mediate it.

Therefore, both positively and negatively charged NPs can adsorb proteins with an overall negative zeta potential, leading to the so‐called “normalization effect”—zeta potential of NPs immersed into the biological media becomes negative no matter what was the surface charge of the pristine NPs (Arcella et al., 2018; Cai et al., 2020; Docter et al., 2015; Emer & Cardoso, 2017; Giulimondi et al., 2019; Gräfe et al., 2019; Srivastava et al., 2018, 2020; Tenzer et al., 2013; Yang et al., 2020).

#### 2.3.2 Neutral and low‐fouling nanomaterials

In 2000, Whitesides et al carried out a systematic analysis of the adsorption of two different proteins (fibrinogen and lysozyme) on the surface of more than 50 self‐assembled monolayers each presenting a different functional group (Chapman et al., 2000). The data allowed the team to propose a set of criteria required to obtain low‐fouling materials, where the term refers to the property of the surfaces to resist the adsorption of proteins and adhesion of cells. These were (i) a high degree of hydrophilicity; (ii) the presence of hydrogen bond acceptors; (iii) the absence of hydrogen bond donors; and (iv) overall neutral charge. The observation that neutral surfaces attract less proteins, compared to charged particles was also supported later by a large number of data sets (Abstiens et al., 2019; Debayle et al., 2019; Digiacomo et al., 2020; Giulimondi et al., 2019; Srivastava et al., 2020; Yang et al., 2020). Besides, Whitesides criteria highlighted how surface charge was not the only factor determining the PC, but that the chemical structure also played an important role, a finding that was subsequently confirmed by others (Arcella et al., 2018; Cai et al., 2020; Mosquera et al., 2020; Yang et al., 2020). An interesting example is a work reported by Sakulkhu et al., using polyvinyl alcohol‐coated superparamagnetic iron oxide NPs, where more proteins adsorbed on the neutral particles covered with hydroxyl groups, than anionic surfaces (Sakulkhu et al., 2015). In this case, the particle surface was enriched with hydrogen bond donors and, as was shown by Chapman et al. by using the example of fibrinogen, the elimination of hydrogen bond donor moieties can significantly reduce the adsorption of proteins (Chapman et al., 2000).

Interestingly, several recent studies have demonstrated that overall surface neutrality is not necessary for low‐fouling properties, if zwitterionic (ZW) materials are used. The term polyzwitterion describes polymers that possess both cationic and anionic groups; these materials display an overall neutral surface charge when placed near the isoelectric point, however, the neutrality is lost if the pH in the solution is different from pI (Lowe & McCormick, 2002). It was observed that ZW gold NPs coated with mixed charged self‐assembled monolayers of –S‐(CH_2_)_10_‐SO_3_^−^ or –S‐(CH_2_)_10_‐NH_3_^+^ showed low‐fouling properties in a wide charge range from −5 to −15 mV (Liu et al., 2012). In addition, a recent study reported the five‐fold reduction in the amount of adsorbed proteins for gold NPs with ZW ligands attached to the surface, even though the overall zeta potential was positive (ξ = +32 mV; Mosquera et al., 2020). This discrepancy between anti‐fouling properties observed for charged materials and Whitesides' criterion about surface neutrality, led to the re‐evaluation of the NPs' characteristics required to limit protein adsorption. Shao proposed a new set of criteria for anti‐biofouling properties (Shao, 2019) specifically for ZW materials, highlighting the important role of the hydration shell, and its ability to suppress electrostatic protein adsorption, a finding subsequently confirmed also by Liu, Zhang, et al. (2020). This feature has been found to play a key role not only for ZW NPs but in general for nonfouling materials (Cao & Jiang, 2012; Debayle et al., 2019; Liu, Zhang, et al., 2020; Scheffer et al., 2020; Shao, 2019; Weiss et al., 2019). For instance, the low‐fouling properties of PEG‐coated materials (Alberg et al., 2020; Meesaragandla et al., 2020; Srivastava et al., 2020) are the result of the hydrations layer due to hydrogen bonds with water molecules (Liu, Zhang, et al., 2020). The anti‐fouling properties of ZW ligands have often been compared to PEG, used as a standard low‐fouling material (Cao & Jiang, 2012; Debayle et al., 2019; Liu, Zhang, et al., 2020; Mosquera et al., 2020; Scheffer et al., 2020; Yang et al., 2014). For example, Liu et al. used a lysosome adsorbed on the surfaces covered with PEG and different ZW ligands, to demonstrate that low‐fouling properties are stronger with a higher level of hydration, which leads to desorption of proteins from the surface due to repulsive forces (Liu, Zhang, et al., 2020). Overall, they have shown that ZW coating is superior to PEG in hydration and resistant to protein adsorption, which can explain the higher blood retention of these nanomaterials in vivo (Perng et al., 2019).

Many different ZW materials have shown low‐fouling ability, however, the extent of this property and the strength of the hydration layer depends on the structure of the ZW ligands. In addition to ensuring low‐fouling characteristics, the structure of the outer layer can play an important role in determining the type of application that a specific nanomaterial can be used for. For example, –COOH functional groups of poly(carboxybetaine methacrylate) can be applied for immobilization or an attachment of targeting ligands, while some other low‐fouling materials—poly(sulfobetaine methacrylate), phosphorylcholine, oligo(ethylene glycol), or PEG‐ do not possess these functionalities (Zhang et al., 2006). As a result, there is a library of low‐fouling coatings with different structures that can be used for specific applications. These include ligands with ZW properties—based on trimethylamine N‐oxide (Li et al., 2019), carboxybetaine, starch (Zhang et al., 2013), hyaluronic acid/chitosan (Almalik et al., 2017), and those with no ZW characteristics—based on citric acid, poly(isobutylene‐alt‐maleic anhydride), poly(sulfonatophenyl)phenylphosphine (Johnston et al., 2017), poly(N‐2‐hydroxypropylmethacrylamide) or polysarcosine (Alberg et al., 2020), to list a few.

#### 2.3.3 Surface charge of NPs and biological response

The inert nature of PEG coating is sometimes correlated with low targeting efficiency and low cellular uptake of NPs (Alberg et al., 2020), the phenomenon is referred to as “PEG dilemma”. Similar problem was also observed in several studies for neutral ZW nanomaterials. For example, ZW lipids‐based liposomes with the low absolute value of zeta potential showed reduced cellular uptake (Yang et al., 2020), while neutral sulfobetaine‐coated QDs—inefficient cellular internalization owing to their overall surface neutrality (Muro et al., 2010). Besides, biologically active groups (amino, mercapto, or carboxylic functionalities) grafted on the surface of silica NPs for targeting purposes were shown to be shielded by ZW ligands attached to the same surface (Loiola et al., 2019). Therefore, despite beneficial low‐fouling characteristics of ZW materials, they not always show improved performance in vivo, however, some studies demonstrate quite promising results. For example, transferrin‐functionalized virus‐like ZW particles were shown to retain high targeting ability in a serum‐rich environment (Zackova Suchanova et al., 2020). Similarly, cysteine‐coated silica NPs with biotin employed as a targeting molecule expressed significantly higher cellular uptake by active targeting in contrast to non‐ZW coatings (Safavi‐Sohi et al., 2016). Very interesting results on cellular internalization were obtained for gold NPs coated with ZW ligands based on complexes between pyranines and positively charged supramolecular cages (Mosquera et al., 2020). Strong positive charge (+32 mV) stimulates dramatic increase in cellular uptake due to passage through negatively charged cellular membrane. The fact that positively charged NPs have high cellular uptake is well known, but the tendency to high protein adsorption of these materials has so far limited their application in vivo. At the same time, positively charged ZW materials offer a promising alternative, since they can support a strong hydration layer around NPs and preserve low‐fouling characteristics together with high cellular uptake.

The trend illustrated in Figure 4, does not apply only to the amount of adsorbed proteins, but also to other biological responses, both in vitro and in vivo. Several publications, for example, have reported a similar trend with regards to the cytotoxicity of nanomaterials. Gold NPs covered with cationic self‐assembled monolayers of –S‐(CH_2_)_10_‐NH_3_^+^ were shown to be more cytotoxic than those with anionic outer monolayers of –S‐(CH_2_)_10_‐SO_3_^−^, while mixed charged coatings showed the lowest cytotoxicity (Liu et al., 2012). Comparable results were recorded for silica NPs, when cytotoxicity and hemolysis occurred for pristine and amine‐functionalized nanomaterials, but not for those covered with ZW moieties (Affonso De Oliveira et al., 2018). Higher cytotoxicity of NPs can be partially attributed to protein denaturation, which was shown to be more significant for positively charged nanomaterials. It was shown by Meesaragandla et al. in a recent study, that neutral gold NPs coated with PEG‐OCH_3_ do not induce changes in the structure of HSA, while positively charged NPs coated with PEG‐NH_2_ induce conformational changes under all pH conditions (Meesaragandla et al., 2020). This study also reported that negatively charged ligands induce conformational changes in HSA at a certain pH, depending on their chemical structure: acidic and physiological pH for citrate‐coated NPs, and acidic and basic pH for PEG‐COOH ligands. Additionally, they also highlight the importance of hydrogen bonding, which can also lead to protein denaturation, as was observed for glycan‐coated NPs at both acidic and basic pH.

In addition to cytotoxicity, charge was also shown to influence the uptake of NPs by macrophages—charged surfaces showed a higher uptake than neutral (Yang et al., 2014), while ZW NPs displayed strong resistance to phagocytic uptake. For example, gold NPs coated with mixed charged monolayers of –S‐(CH_2_)_10_‐NH_3_^+^ and –S‐(CH_2_)_10_‐SO_3_^−^ in different ratios (ξ ranging from −5 to −15 mV) had 7–13 times less uptake by phagocytes than NPs, coated only with –S‐(CH_2_)_10_‐SO_3_^−^ and 25–50 times lower uptake than cationic ones coated with only –S‐(CH_2_)_10_‐NH_3_^+^ (Liu et al., 2012). The reason for the higher uptake by macrophages can be found in the preferential adsorption of opsonins (Gessner et al., 2003; Giulimondi et al., 2019). An adsorption of such opsonin as IgG was shown to be higher on surfaces bearing stronger basic functional group, therefore, stronger positive charge, while a dysopsonin ApoH was found enriched on the negative surface (Gessner et al., 2003). Similarly to PEG, the surface of ZW nanomaterials have shown an enrichment with dysopsonin clusterin (Srivastava et al., 2020), which leads to lower uptake of ZW materials by macrophages and their stealth properties (Encinas et al., 2019; Li et al., 2019; Liu et al., 2012).

Overall, literature data suggest that positively charged non‐zwitterionic NPs show the highest protein adsorption and the most challenging performance in vivo. The use of these nanomaterials for drug delivery application should be carried out with caution, since these coatings can induce several undesirable effects, such as protein denaturation, cytotoxicity, high uptake by macrophages, leading to fast clearance of NPs from the body. Negatively charged mono‐ligand NPs display slightly lower adsorption of proteins, but, still, they are not as effective as neutral materials in terms of low‐fouling characteristics and beneficial biological response. ZW ligands can be considered promising candidates for coatings of NPs, since new interesting data keep emerging regarding not only their low‐fouling characteristics, but also the ability to be used for targeting purpose. Especially, interesting area of research where just a few studies but with promising results were published is related to the use of non‐neutral mixed‐charged NPs. These materials can use the benefits of charged surfaces (e.g., interactions with cellular membrane), but at the same time, strong hydration can prevent protein adsorption and undesirable biological response related to that.

## 3 PROTEIN CORONA AS A FUNCTION OF MORPHOLOGY

With the advances achieved in the area of nanomaterials, it is not surprising that research on PC formation focused extensively on the understanding of the impact of NPs' morphology. The next section provides an overview of the most recent findings, focusing on size, shape, and particles' surface roughness.

### 3.1 Nanoparticle size

**FIGURE 5:**
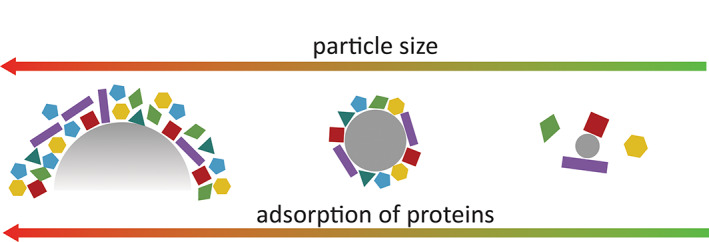
Schematic representation of the effect of particle size on the formation of protein corona

Proteins are known to adsorb on the surface of biomaterials (Nakanishi et al., 2001), and, with their high surface‐to‐volume ratio, nanomaterials would therefore be expected to adsorb higher quantities of proteins. The team led by Dawson pioneered research in this area, focusing initially on the effect of size on the adsorption of proteins onto pNIPAM‐BAM nanogels (Cedervall et al., 2007). Their data suggested that smaller particles (size <70 nm) showed lower affinity to HSA compared to larger particles (size around 200 nm), a fact that is somewhat counterintuitive. This finding, illustrated by Figure 5, was then demonstrated with various NP systems, such as polystyrene NPs (Lima et al., 2020), dendrimers (Pant et al., 2017), silica NPs (Marichal et al., 2020), and metal NPs (Casals et al., 2010; De Paoli Lacerda et al., 2010; Yu et al., 2017) Despite the higher surface/volume ratio, small NPs present a more curved surface compared to larger ones. There is consensus among scientists that the highly curved surfaces are less accessible to proteins due to steric effects caused by protein overcrowding and a lower surface area available for individual proteins.

More recently, the size of NPs was also found to control the thickness of the PC formed on gold nanospheres. Piella et al were able to synthesize gold NPs with increasing particles sizes, from 3.5 to 150 nm and measure the thickness of their PC in cell culture media using dynamic light scattering (DLS; Piella et al., 2017). As expected, particles with larger sizes were able to adsorb more proteins on their surface compared to the smaller ones. As a result of the range of particles sizes investigated, it was possible to observe that the thickness of the PC did not increase linearly with particle size, resulting in three distinct areas within the corona. Particles with size <10 nm formed an incomplete layer of proteins, which did not cover completely the surface of the gold NPs; a complete layer of proteins was observed with NPs' size ranging from 10 to 80 nm, while size >80 nm led to the formation of multiple layers of proteins, known as multilayer corona. This interesting study provides initial evidence of the role that particle size plays not only in determining the quantity of proteins adsorbed, but also the structure of the formed PC. Measurements of the PC's thickness so far were carried out on the NP‐complex after isolation from the biological media, focusing the analysis on the hard corona. Further investigation will be needed to determine the effect of particle size on the formation of the soft corona that is lost during isolation of the complex. This new knowledge could provide a useful tool to control the formation of the PC and in future tailor its properties depending on the specific applications.

When investigating PC formation, one of the issues is the conformation of the adsorbed proteins. Denaturation of proteins, often induced by their adsorption on the NP surface, has been reported and shown to affect the interaction of the nanomaterials with cells in vitro (Liu et al., 2021; Yan et al., 2013), and NP's size plays a role. De Paoli Lacerda et al. used fluorescence quenching and circular dichroism to show that gold NPs with larger particle size caused more significant conformational changes in the structure of the model protein histone H3 (De Paoli Lacerda et al., 2010). In a more recent article from 2019, Goy‐Lòpez et al. used a similar approach involving fluorescence quenching analysis to demonstrate that gold NPs with larger diameter were also capable to cause stronger denaturation in the structure of human albumin (Goy‐Lòpez et al., 2012). The same trend was then observed with silica NPs by Satzer et al, who found that both BSA and myoglobulin were significantly denatured, when adsorbed in the corona of particles with size >150 nm (Satzer et al., 2016). Collectively, these studies on gold NPs and silica NPs, provide evidence that larger particles cause a more significant denaturation of the PC. This effect also plays an important role in the design of nanomaterials and in the ability to predict their physicochemical properties in biologically relevant media. For example, the presence of exposed hydrophobic regions in the structure of denatured proteins may facilitate further adsorption of proteins, thus, enriching the already existing PC and mediating the formation of a multilayer corona (Meersman & Heremans, 2003).

Although the effect of particle size on the number of proteins adsorbed on the PC have been widely reported, the effects of particle size on the composition of the PC, especially on the diversity of proteins, are still the object of interesting studies. A pioneering work by Lundqvist et al. showed that neutral polystyrene NPs, with a size 50 or 100 nm, led to the formation of a highly homologous PC (homology >80%; Lundqvist et al., 2008). However, in the case of amine‐ or carboxyl‐functionalized polystyrene particles, the formed corona presented much lower level of homology (50%) between 50 and 100 nm size, suggesting that surface chemistry, and not NP size, plays a major role in determining PC composition. Shortly after, Dobrovolskaia et al. observed a similar trend, with gold NPs of smaller diameter (30 nm) forming a more diverse corona, by adsorbing 48 different proteins, compared to only 21 obtained with larger (50 nm) gold NPs (Dobrovolskaia et al., 2009). More recently the role of NP's size and surface chemistry in influencing PC diversity has attracted renewed interest. In 2020, Lima et al. found that polystyrene NPs with smaller size (26 nm) adsorbed a total of 6 unique protein species compared to the 83 and 49 found on particles of 80 and 200 nm, respectively (Lima et al., 2020). However, this is in contrast to the data reported by Marichal et al. in the same year, who found that the composition of the PC formed on silica NPs with different sizes was overall the same (Marichal et al., 2020). This could be the result of the different chemical composition of the NPs, or due to the protocols used. The analysis of the PC is generally carried out on the isolated NP‐protein complex (Böhmert et al., 2020), with the inevitable loss of the low‐affinity proteins, which could artificially increase the degree of corona similarity (Winzen et al., 2015). There is a lack of consistency in the literature over the conditions used during the isolation step, often tailored to specific materials and, thus, hardly comparable. Therefore, it is possible that the development of a “softer” isolation process and the introduction of alternative isolation techniques such as field‐flow fractionation will provide more consistent and comparable data.

It is important to note that the strong interest in the use of nanomaterials for therapeutical applications has currently driven the research toward studies using human or mouse plasma and protein extracts from biological media, where globular structures are prevalent. As the interests in this area widen, the study of the formation of PC as a result of interactions with membranes and other nonglobular proteins is expected to broaden our understanding.

### 3.2 Nanoparticle shape

Recent developments of new synthetic methodologies for NPs' synthesis have allowed a systematic investigation of the effects of NPs' shape on their properties (Wu et al., 2016), providing a potential tool to tailor the behavior of NPs both in vitro and in vivo (Da Silva‐Candal et al., 2019; Zhao et al., 2017), however, the role that shape can play in influencing PC formation on NPs has yet to be fully explored.

In 2018, García‐Alvarez et al. demonstrated that for gold NPs, particle shape could influence the formation of a PC in vivo (García‐Álvarez et al., 2018). Rod or star‐shaped gold NPs with a diameter of either 40 or 70 nm were injected in mice and the corona‐coated particles were isolated from the animal's plasma through a previously validated chromatographic method (Hadjidemetriou et al., 2016). Total amount of proteins recovered from stars were significantly higher compared to rods of the same size, indicating that NPs shape significantly altered the affinity of the particles for serum proteins. Protein qualitative analysis via LC–MS/MS revealed that the composition of the PC was overall relatively similar for 70 nm NPs, with stars and rods sharing 82% and 72% of the proteins in their corona respectively. PC diversity increased with 40 nm NPs instead, with gold nanostars having a slightly more diverse corona (about 64% of common proteins) compared to gold nanorods (88%). This study features a comprehensive analysis of PC obtained from in vivo conditions and offers therefore a representative view on the effect of particle shape on the formation of PC.

More recently, particle shape was also found to affect the formation of PC on mesoporous silica nanoparticles (MSN). Visalakshan et al were able to show that the amount of proteins adsorbed on rods from serum was higher (6 mg/m^2^) compared to spheres (1.5 mg/m^2^; Visalakshan et al., 2020). Notably, the particle shape directly affected the composition of the adsorbed corona and in particular the relative amount of immunoglobulins, which were found to be 57% of the total corona in the case of rods as opposed to 42% for spheres. This result suggests that although protein adsorption on unfunctionalized particles is generally driven by unspecific interactions, certain particle shapes may favor the adsorption of specific proteins and, possibly, affect the fate of nanomaterials in vivo.

The shape of MSN has also been found to directly affect the conformation of adsorbed proteins. When incubated with rod or spheres MPS, the conformation of model proteins HSA, fibrinogen, and IgG was found to be affected (Ma et al., 2014). Although rods were found to have a stronger effect on the secondary structure of HSA, fibrinogen was denatured to a higher degree by spherical particles instead, showing that for a given particle shape, only certain proteins in the corona may undergo denaturation. Evidence of denaturation of certain proteins in the corona could help to elucidate the role of particle shape in determining the biological behavior on particles in vitro and in vivo.

The hypothesis that NPs with similar chemical properties but the different shapes could lead to varied responses at cellular level, due to inherently different PC, stimulated further research. Kong et al. evaluated the effect of the shape of Fe_3_O_4_ NPs and their PC on cellular response focussing on how NPs affected the levels of cellular autophagy, an important process with implications in cellular homeostasis (Kong et al., 2018). Interestingly, levels of autophagy induced by the particles were affected differently by the PC depending on the particle shape. Both cubes and rods registered a significant increase in the levels of autophagy (3‐fold increase for rods, 0.5‐fold increase for cubes) consistent with higher amounts of proteins bound to rods compared to cubes, confirming the role that shape plays in corona formation. Interestingly, spherical particles showed an opposite trend, with levels of autophagy decreasing in the presence of proteins.

Although current literature has so far mainly focussed on inorganic NPs, evidence suggests that shape may play a role in the formation of PC and in determining both its physicochemical and biological properties. The challenges associated with the synthesis of nanomaterials with well‐defined shapes still hinder the extensive investigation of this parameter. The low number of comprehensive reports involving a large variety of particle shapes still precludes the formulation of general rules that can be applied in the process of designing novel nanomaterials. Nevertheless, the possibility of exploiting particle shape to tailor the properties of PC and its impact on biological entities represents an interesting opportunity to develop more effective nanomaterials for biomedical applications.

### 3.3 Surface roughness

Scientists have recently started to look at additional properties that may influence PC formation, and among these NPs' roughness has led to interesting results. Piloni et al evaluated the effect of surface roughness focussing on self‐assembled micelles (Piloni et al., 2019). Micelles of around 100 nm in size and with either smooth or rough surfaces were prepared from the self‐assembly of linear ABC triblock co‐polymers comprising a sugar‐functionalized hydrophilic block, a hydrophobic block, and a third block controlling the morphology of the micelle during self‐assembly. After incubation in serum‐supplemented medium, micelles presenting smooth surface showed formation of a PC by DLS and quartz crystal microbalance, whereas micelles with rough surface formed no or little PC instead. With their irregular surface, “rough” micelles display increased local surface curvature, making the binding of proteins to the surface of the particles more difficult compared to smooth micelles.

A similar approach, studying the effect of surface roughness on PC formation, was used by Xia et al. although 150 nm gold NPs with either smooth or rough surfaces were chosen for this work (Xia et al., 2020). DLS, zeta potential, and isothermal titration calorimetry were employed to offer a comprehensive characterization of the complex formed by these two different types of particles and the model protein BSA. Overall, rough particles displayed lower affinity for albumin compared to smooth ones, suggesting that surface morphology can play a role in the formation of PC in inorganic nanomaterials as well. Importantly, additional circular dichroism analysis was carried out to further investigate the effect of surface roughness on the secondary structure of BSA adsorbed on the particle's corona. Interestingly, gold NPs with a rough surface caused a stronger denaturation of BSA, indicating that the adsorption of a protein on a rougher surface may lead to stronger protein denaturation in comparison to a smooth one. The higher reactivity of uncoordinated gold atoms found on the highly curved surface of rough NPs may lead to stronger binding of albumin possibly via gold–sulfur covalent bonds, forcing a stronger denaturation in the adsorbed protein. Although the mechanism proposed by the authors might apply only to gold and other inorganic NPs, this is an important evidence of the dual role played by NP's surface roughness, in limiting PC while enhancing protein denaturation. This highlights the importance of considering multiple factors, when tailoring the design of NPs, to avoid introducing undesired properties.

**FIGURE 6:**
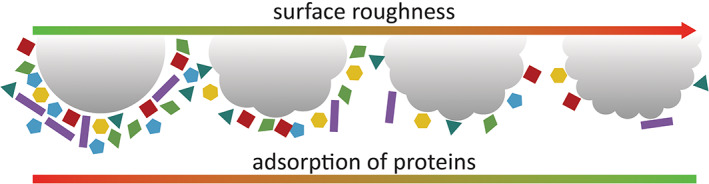
Schematic representation of the effect of particle surface roughness on the formation of protein corona

The study of the effect of surface roughness was more recently extended to polystyrene microspheres, as an example of organic particles. Chen et al. adopted a method involving particle surface etching via chromic acid treatment that allowed the preparation of polystyrene particles with five different degrees of surface roughness, estimated by scanning electron microscopy analysis, depending on the etching protocol conditions (Chen et al., 2020). When particles were incubated with either plasma or single proteins such as BSA and fibrinogen, both the total adsorbed amount and the overall corona composition were found to be affected by the etching procedure. As shown in Figure 6, smooth‐unetched microspheres were found to adsorb the highest number of proteins, whereas all etched particles showed overall lower adsorption of proteins. Thanks to the larger set of particles investigated, a direct correlation between low protein adsorption and increasing number of surface defects on the particles (degree of surface roughness) could be drawn. Further analysis using SDS‐PAGE and LC–MS on plasma incubated polystyrene microparticles also showed differences in the composition of PC of rough particles compared to smooth ones. As the composition of the PC may affect the interaction of particles with cells, in vitro macrophage phagocytosis of differently etched particles in the presence of serum was evaluated. Remarkably, microspheres with increasing surface roughness were associated with higher degree of phagocytosis, indicating that surface roughness of the particle may be tuned to control the degree of cellular internalization of the particles in macrophages. This work provides important evidence suggesting that the degree of PC formation may be tuned by gradually changing the roughness of particles and that this may be used to control the behavior of the nanomaterials in vitro.

NPs' morphology significantly affects many properties of the PC, such as number of proteins adsorbed, degree of protein denaturation, and overall PC composition. Nanomaterials with small diameters and rough surfaces may be designed to hinder the adsorption of proteins and retain their original surface chemistry even in protein‐rich environment. However, particles with larger sizes, smooth surfaces, and nonspherical shapes might be synthesized to form a PC and improve their toxicological profile or enhance their recognition by cells of the immune system. The development of novel approaches to control the morphological properties of nanomaterials, will allow to finely tune the properties of the PC, leading to exciting new approaches to harvest the full potential of the PC.

## 4 CONCLUSION AND OUTLOOK

The hydrophobic/hydrophilic character of the NPs has been shown to significantly influence both the quantity and the composition of the protein adsorbed on the NPs surface. Overall, more hydrophobic NPs are characterized by more enhanced protein adsorption. This trend, which is evidence of the primary role played by hydrophobic forces in the PC formation, has been observed in the case of both organic and inorganic nanomaterials. For such reasons, chemical modifications of NPs with hydrophilic motifs such as PEG and other equivalents have been broadly used to reduce interactions with proteins and therefore improve their behavior in vivo. However, the importance of controlling the type of proteins adsorbed has been highlighted more recently, and nanomaterials interacting prevalently with dysopsonins rather than with opsonins have shown the best correlation between properties and application. The steric hindrance and the chirality of NPs' surface is known to impact PC formation, however, the identification of specific points of contact requires specialized techniques complemented by computer simulations. On the other hand, the presence of charges can significantly impact protein adsorption, with positive non‐zwitterionic showing the highest and neutral zwitterions displaying the lowest, allowing for the much sought after low‐fouling properties. Recent advances in synthetic methodologies have allowed the investigation of the role that shape plays in PC formation, although current results are available mainly for inorganic NPs. This has also been enhanced by very interesting studies on surface roughness. The limited data available have not yet allowed to fully explore the impact of these parameters and to identify trends, however, we envisage these to be key parameters of great future interest, for the development of more effective nanomaterials for biomedical applications.

In the last few years, the adsorption of a wider range of biomolecules on the surface of NPs has been studied. By coupling chromatographic methods and mass spectrometry, nanomaterials have been shown to adsorb small molecules such as lipids (Gunnarsson et al., 2018; La Barbera et al., 2020; Lee et al., 2018; Lima et al., 2020), amino acids (Pink et al., 2018) and oligonucleotides (Vidaurre‐Agut et al., 2021), with lipids being nowadays the most investigated. In a recent study, Chetwynd et al. demonstrated that, similarly to PC, the corona of metabolites can vary significantly between silica, TiO_2_, and polystyrene NPs, indicating a possible role of surface chemistry of materials on their interactions with small molecules (Chetwynd et al., 2020). Although the metabolite corona has been so far mainly analyzed from a qualitative and quantitative point of view, initial investigations by Emer et al. suggest that even in the absence of proteins, surface charge, colloidal stability, and antibacterial properties of silica NPs were altered in the presence of nutrients‐rich cell media (Emer & Cardoso, 2017). It is reasonable to expect that a metabolite corona may affect physical–chemical properties of several other nanomaterials, thus prompting for more future characterization of this phenomenon. Another exciting and recent evolution in the field of PC, is the interaction between the protein and the metabolite coronae to form the biomolecular corona. Recent work from Lima et al. strongly suggests that the adsorption of lipids on polystyrene NPs may be mediated by their interaction with apolipoproteins located on the surface of lipid vesicles (HDL) responsible for lipids transport through the body (Lima et al., 2020). This work indicates that protein and metabolite coronae are able to influence each other and that integrated analysis of the different components of the biomolecular corona may be pivotal to gain a better and broad understanding of the PC.

The refinement of strategies to tailor PC formation and composition together with a better understanding of the impact of each structural parameter will allow not only to enhance NPs bioavailability and potential for biomedical applications but also could be further used to promote specific biological activities.

## CONFLICT OF INTEREST

The authors have declared no conflicts of interest for this article.

## AUTHOR CONTRIBUTIONS

**Roberta Bilardo:** Conceptualization (equal); data curation (equal); writing – original draft (equal). **Federico Traldi:** Conceptualization (equal); data curation (equal); writing – original draft (equal). **Alena Vdovchenko:** Conceptualization (equal); data curation (equal); writing – original draft (equal). **Marina Resmini:** Conceptualization (lead); formal analysis (supporting); funding acquisition (lead); supervision (lead); writing – original draft (equal); writing – review and editing (lead).

## RELATED WIREs ARTICLES

Protein corona and nanoparticles: How can we investigate on?

Impact of the protein corona on nanomaterial immune response and targeting ability

## Data Availability

Data sharing is not applicable to this article as no new data were created or analysed in this study.
